# Tricuspid regurgitation in the era of transcatheter intervention: beyond multivalvular complexity towards haemodynamic phenotyping

**DOI:** 10.1093/eschf/xvag154

**Published:** 2026-05-28

**Authors:** Erwan Donal, Vincent Auffret, Guillaume L’Official, Guillaume Leurent

**Affiliations:** Cardiology Department, University of Rennes, CHU Rennes, Inserm, LTSI—UMR 1099, 2 rue Henri le Guilloux, Rennes F-35000, France; Cardiology Department, University of Rennes, CHU Rennes, Inserm, LTSI—UMR 1099, 2 rue Henri le Guilloux, Rennes F-35000, France; Cardiology Department, University of Rennes, CHU Rennes, Inserm, LTSI—UMR 1099, 2 rue Henri le Guilloux, Rennes F-35000, France; Cardiology Department, University of Rennes, CHU Rennes, Inserm, LTSI—UMR 1099, 2 rue Henri le Guilloux, Rennes F-35000, France

**Keywords:** Tricuspid regurgitation, Transcatheter intervention, Multivalvular disease, Phenotyping, Trans-catheter edge-to-edge

The recognition of tricuspid regurgitation (TR) as a major determinant of morbidity and mortality represents one of the most important paradigm shifts in contemporary valvular heart disease.^1,2^ Once considered a secondary and largely benign consequence of left-sided heart disease, TR is now understood as a major contributor to right-sided heart failure, systemic congestion, and adverse outcomes. Simultaneously, the emergence of transcatheter tricuspid valve interventions—particularly transcatheter edge-to-edge repair (T-TEER)—has profoundly altered the therapeutic landscape, offering treatment opportunities to a population historically deemed too high risk for surgery.^2–7^

In this context, the EuroTR registry analysis provides timely and clinically relevant insights into the complexity of patients undergoing T-TEER in contemporary real-world practice, focusing specifically on the impact of concomitant left-sided valvular heart disease.^8^

## Multivalvular disease: the rule rather than the exception

The EuroTR investigators report data from 1647 patients undergoing T-TEER, showing that concomitant left-sided valvular heart disease is nearly ubiquitous, with more than one-third presenting with at least moderate disease. This observation is highly informative. Randomized trials, by necessity, often enrol selected populations and exclude patients with substantial concomitant pathology. Real-world registries reveal the true complexity of patients encountered in daily practice.^8^

Importantly, the presence of at least moderate left-sided valvular disease was independently associated with worse survival and higher rates of heart failure hospitalization at 2 years. These findings align with current understanding that multivalvular disease often reflects advanced global cardiopulmonary involvement, characterized by elevated filling pressures, pulmonary hypertension, right ventricular (RV) dysfunction, and progressive systemic congestion.^9,10^

Yet the most interesting finding may be that despite this unfavourable baseline profile, T-TEER achieved significant TR reduction and clinically meaningful symptomatic improvement across subgroups. Worse prognosis did not preclude therapeutic response.

That distinction matters.

## Rethinking causality: tricuspid regurgitation as a driver, not a bystander

The traditional pathophysiological framework for TR has largely been linear. Left-sided valvular disease increases left atrial pressure, leading to pulmonary hypertension, RV remodelling, annular dilation, and ultimately TR. In that model, TR is simply the downstream consequence of upstream pathology.

The EuroTR findings only partially support that interpretation.^8^

Patients with concomitant left-sided disease indeed exhibited more advanced remodelling and higher pulmonary pressures. However, the persistence of symptomatic benefit following TR correction—even in these complex patients—strongly argues against viewing TR as merely an epiphenomenon.

A more integrated model is needed. Tricuspid regurgitation contributes directly to systemic venous congestion. Congestion impairs renal and hepatic function. Reduced forward stroke volume worsens low-output physiology. Right ventricular inefficiency further compromises interventricular interaction and left-sided filling. The result is a self-reinforcing haemodynamic spiral.^10,11^

Within this framework, TR is not simply the consequence of disease progression. It becomes an active participant in disease progression. This conceptual shift has important clinical consequences.

## The added value of randomized evidence

Registry analyses provide essential external validity but remain inherently observational.^8^ Associations between multivalvular disease and adverse outcomes may reflect confounding by disease severity, referral bias, or comorbidity burden rather than direct causal mechanisms.

Randomized evidence is therefore critical.^3–5^

The Tri.Fr trial represented an important milestone by demonstrating that T-TEER plus optimized medical therapy improved quality of life compared with optimized medical therapy alone in patients with severe isolated TR. More importantly, emerging longer-term evidence suggests that correction of severe TR may influence clinical trajectories beyond symptom relief alone, particularly regarding heart failure burden.^3,7,12^

This context matters when interpreting EuroTR. The registry should not be read as suggesting diminished efficacy of T-TEER in complex patients. Quite the opposite. It identifies a higher-risk phenotype that still derives meaningful benefit from intervention.^8^

The message is not therapeutic futility. The message is delayed intervention in advanced disease.

## From anatomical classification to haemodynamic phenotyping

A key limitation of the EuroTR analysis lies in the categorization of patients according to anatomical descriptors rather than true physiological profiling.

The label of ‘at least moderate left-sided valvular disease’ encompasses highly heterogeneous entities: functional mitral regurgitation driven by atrial remodelling, degenerative mitral disease, calcific aortic stenosis, and mixed lesions with varying haemodynamic relevance.

These conditions are biologically and prognostically distinct. Grouping them under a single anatomical umbrella risks obscuring the mechanisms truly driving outcomes.^13,14^

Emerging evidence suggests that prognosis in TR is determined less by the enumeration of valve lesions and more by integrated haemodynamic status.

Key determinants likely include RV–pulmonary artery coupling, residual TR burden, right atrial remodelling, venous congestion severity, hepatic and renal dysfunction, and forward flow reserve.^12,13^

These parameters are accessible in contemporary practice, particularly through comprehensive echocardiographic assessment.^8–10^

A haemodynamic phenotyping approach may therefore offer greater clinical precision than purely anatomical classification. Concomitant left-sided disease may simply represent a surrogate marker of advanced cardiopulmonary decompensation rather than the primary determinant of adverse outcomes.

## Clinical implications

Several practical implications emerge. First, multivalvular disease should not be viewed as a contraindication to tricuspid intervention. The symptomatic benefit observed across subgroups supports continued intervention even in clinically complex patients.

**Figure 1:**
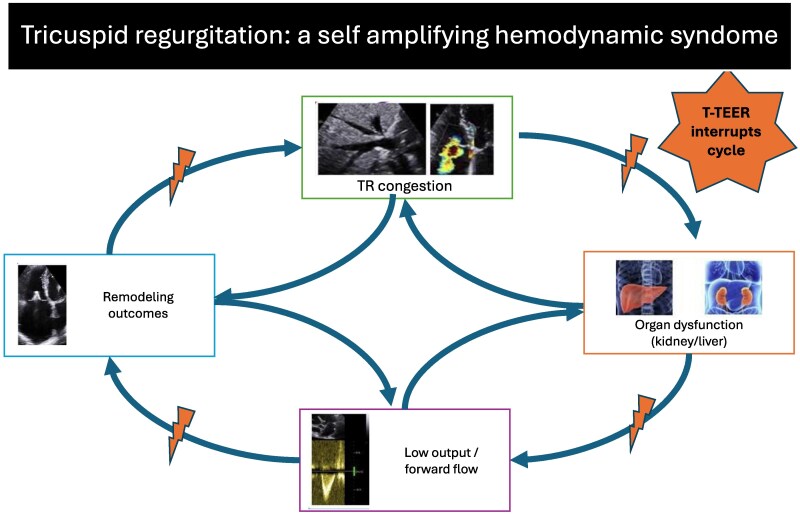
Summary

Second, risk stratification must evolve beyond valve-centric thinking (*Figure 1*).

The historical approach to valvular heart disease has often focused on lesion severity in isolation. That model is increasingly insufficient in TR. A patient with torrential TR but preserved RV function and limited congestion is fundamentally different from a patient with moderate residual TR, severe RV dysfunction, progressive end-organ impairment, and exhausted forward flow reserve.^6,13^

Phenotype matters more than anatomy alone. Third, timing is likely crucial.^14^

The poorer outcomes observed in patients with concomitant left-sided disease likely reflect treatment delivered too late in the disease course, after substantial RV dysfunction and systemic decompensation have already developed. Intervening earlier—before irreversible right heart failure—may prove more important than increasingly sophisticated rescue strategies in advanced disease.^6^

## Bridging the gap between trials and real-world practice

The evolution of tricuspid intervention mirrors the broader trajectory of structural heart disease therapies. Randomized trials establish internal validity and define therapeutic efficacy in selected populations. Registries provide external validity and reveal the complexity of daily clinical practice.

These evidence streams should not be positioned in opposition.

They are complementary. Trials such as Tri.Fr and TRILUMINATE establish that correcting severe TR improves clinically meaningful outcomes. Registries such as EuroTR define the heterogeneity of the population now being referred for intervention.^3–5,8^

The challenge moving forward is integration. Patient selection should no longer rely solely on anatomical grading. Decision-making should increasingly incorporate markers of congestion, RV adaptation, forward flow competence, and right-sided reserve.

That transition—from anatomical classification to physiological phenotyping—may represent the next major advance in tricuspid valve intervention.^15^

## A broader conceptual shift

The implications extend beyond TR itself. Multivalvular disease should not be understood simply as the coexistence of multiple lesions. It often reflects a systemic syndrome of chronic haemodynamic overload, maladaptive remodelling, and progressive circulatory failure.

This is why a purely lesion-focused framework becomes limiting. Correcting TR is not merely fixing a valve. It may interrupt a broader haemodynamic cascade.^15^

That perspective aligns with contemporary heart failure thinking, where congestion, ventricular interaction, and reserve capacity increasingly guide management more effectively than isolated structural descriptors (*Figure 1*).

Structural intervention is evolving from anatomy-driven repair towards physiology-guided cardiovascular medicine. Tricuspid regurgitation may become one of the clearest examples of this transformation.

## Conclusion

The EuroTR analysis highlights both the remarkable prevalence and clear prognostic significance of concomitant left-sided valvular heart disease in patients undergoing T-TEER. These findings should not be interpreted as diminishing the value of tricuspid intervention. Instead, they reinforce a more important concept: TR is a central, modifiable component of a complex haemodynamic syndrome. Randomized evidence has already demonstrated that correcting severe TR improves patient-centred outcomes. Real-world evidence now clarifies that the patients we treat are often far more complex than those enrolled in pivotal trials.

The next challenge is refinement. The future of TR management will depend less on counting valve lesions and more on identifying the right physiological phenotype, selecting the right timing, and intervening before irreversible right-sided failure becomes established.

That is where the field should move next.

## Declarations

### Data Availability

No data were generated or analysed for this manuscript.
